# ﻿A new species and record of *Xestochironomus* Sublette & Wirth, 1972 (Diptera, Chironomidae) from the Dominican Republic, with males and females associated by DNA barcode

**DOI:** 10.3897/zookeys.1266.162131

**Published:** 2026-01-12

**Authors:** Lívia Maria Fusari, Amelie Höcherl, Caroline Chimeno, Viktor Baranov

**Affiliations:** 1 Universidade Federal de São Carlos (UFSCar), São Carlos, Brazil Universidade Federal de São Carlos (UFSCar) São Carlos Brazil; 2 SNSB – Zoologische Staatssammlung München, München, Germany SNSB – Zoologische Staatssammlung München München Germany; 3 BeOne Medicines Germany GmbH, Theresienhöhe, München, Germany BeOne Medicines Germany GmbH München Germany; 4 Bavarian State Zoological Collection (SNSB-ZSM), München, Germany Bavarian State Zoological Collection (SNSB-ZSM) München Germany; 5 Estación Biológica de Doñana, Consejo Superior de Investigaciones Científicas (CSIC), Seville, Spain Estación Biológica de Doñana, Consejo Superior de Investigaciones Científicas (CSIC) Seville Spain

**Keywords:** Aquatic insects, DNA barcoding, Neotropical, *Stenochironomus* complex, wood-mining

## Abstract

We describe a new species of *Xestochironomus* Sublette & Wirth, 1972 and provide notes on *X. luteifurcatus* Sublette & Wirth, 1972 from the Dominican Republic. The adult male and female of *Xestochironomus digitulus***sp. nov.** are also described and illustrated. The male and female of *X. luteifurcatus* are redescribed. Males of the new species can be easily distinguished from congeners by the following combination of characters: the abdomen of both sexes bears cross bands; the anal point is long, slender; the male gonostylus has a slightly bifurcated apex; and there is a thumb-like lobe at the apex of the gonostylus.

## ﻿Introduction

The Greater Antilles is an important biogeographical region for understanding the faunal exchange between the Nearctic and Neotropics. This is mostly because of the dispersal of the many organisms by “island hopping long before the formation of the isthmus of Panama and the great biotic interchange during the Miocene–Pleistocene (Viñola-López et al. 2022). The island of Hispaniola provides a great opportunity to understand the biotic interchange between the Neotropical and Nearctic realms due to abundant and species-rich deposits in Dominican amber; this provides a snapshot of the Miocene fauna, which can be compared to that now extant (Grund 2006). Comparison of the modern and extant fauna of Hispaniola provides fertile ground for developing a historical biogeographical framework. One of the groups of the animals studied relatively well in the Dominican amber are non-biting midges (Diptera, Chironomidae) (Grund 2006). Grund (2006) reported the presence in the Miocene fauna of the Dominican Republic numerous chironomids and found them related to the extant wood-mining genera, namely *Stenochironomus* Kieffer, 1919 and *Xestochironomus* Sublette & Wirth, 1972. These records provide useful points of calibration of biogeographic models of chironomid dispersal between the Nearctic and Neotropics. Unfortunately, the extant chironomid fauna of Hispaniola is not well enough known to provide such a point of comparison (Silva et al. 2015; Andersen et al. 2023, 2024). In this contribution, we expand the knowledge of the genus *Xestochironomus* from the Dominican Republic.

Sublette (1967) erected the genus *Insulanus* to accommodate Chironomus (Stenochironomus) furcatus Johannsen, 1938 from Puerto Rico and Costa Rica. However, the name *Insulanus* was preoccupied, and Sublette and Wirth (1972) introduced the replacement name *Xestochironomus* and described an additional six new species from Caribbean islands. In his revision of the *Stenochironomus* complex, Borkent (1984) added five new *Xestochironomus* species from the USA and Venezuela. Later, Sublette and Sasa (1994) described another new species from Guatemala. Andersen and Kristoffersen (1998) described two new species from Chile and Costa Rica, Pinho and Souza (2013) described two new species from Brazil, and González et al. (2016) described another new species from Cuba.

Below, we describe a new species of *Xestochironomus* from the Dominican Republic. Morphologically the species belongs to the group of “*Xestochironomus* with gonostyli forked” (similar to *X. nebulosus* Sublette & Wirth, 1972 from Puerto Rico and *X. naranjoi* González, Andersen & Hagenlund, 2016 from Cuba). The new species can easily be separated from all its congeners, based on the combination of color pattern, particularly that of the legs and abdomen, slender anal point, slightly bifurcated apex of the male gonostylus, and thumb-like lobe at the apex of gonostylus. We have also expanded the knowledge of morphology of the previously described *X. luteifurcatus* Sublette & Wirth, 1972. We sequenced the cytochrome b subunit I (COI) gene of both species was sequenced and added the sequence data to the public databases.

## ﻿Materials and methods

The specimens were collected, preserved in ethanol 90, and later mounted in Euparal following the procedure outlined by Sæther (1969). The morphological nomenclature follows Sæther (1980). Measurements were made according to the method suggested by Epler (1988). Measurements are given as ranges, the number of samples measured is indicated in brackets after the range of measurements. Only when the number of specimens examined is equal to or greater than five, and we provide the average value after the size range. We used Adobe Illustrator for creation of line drawings and Adobe Photoshop for editing photographs.

The specimens were collected under the collection permit from the Ministerio de Medio Ambiente y Recursos Naturales of the Dominican Republic for the project “Long peace of the Caribbean – have the biota of the Dominican Republic really remained virtually unchanged for over 13 million years?”. Specimens were exported under permit #VAPB-07404. The holotype and paratypes were deposited in the collection at Estación Biológica de Doñana (EBD), paratypes will be deposited in the Chironomidae collection of the Aquatic Entomology Laboratory (LEA) of the Universidade Federal de São Carlos and in the collection of Museo Nacional de Historia Natural “Prof. Eugenio de Jesús Marcano” (MNHN-EJM).

DNA was extracted from specimens at the SNSB molecular lab using the NucleoSpin 96 Tissue (Macherey-Nagel) DNA-extraction kit after having undergone an overnight lysis at 56 °C. The COI barcodes were amplified using the LepF1 and LepR1 standard barcoding primers (Leray et al. 2013) using a Biometra Thermocycler (Analytik Jena) and the following PCR conditions: 2 min at 94 °C; first cycle set (5 repeats): 30 s denaturation at 94 °C, 40 s annealing at 45 °C and 60 s extension at 72 °C. Second cycle set (35 repeats): 30 s denaturation at 94 °C, 40 s annealing at 51 °C and 60 s extension at 72 °C; final elongation 10 min at 72 °C. The PCR products were cleaned using the ExoSAP-IT Express (Thermo Fisher) Kit and sent to the LMU Sequencing Service at Biozentrum (Martinsried, Germany) for Sanger sequencing. Every specimen’s COI barcode was sequenced as a forward and reverse strand. The original traces were uploaded as well. The editing and alignment of the sequences were done in Geneious Prime 2023.1.1 build 2023-04-03 (https://www.geneious.com). A tree was constructed using the neighbor-joining method with 1,000 bootstrap replicates and genetic distances were estimated using the Kimura-2-parameter (K2P) model in MEGA v. 12 (Kumar et al. 2024). Sequences are deposited in GenBank under the accession numbers PV761055 to PV761064.

## ﻿Results

### ﻿Taxonomic account

**Family Chironomidae Newman, 1834**

**Subfamily Chironominae Macquart, 1838**

***Xestochironomus* Sublette & Wirth, 1972**

#### ﻿ Xestochironomus digitulus

Taxon classificationAnimaliaDipteraChironomidae

Fusari & Baranov sp. nov.

AD041A30-B62F-561A-A434-D3022736D241

https://zoobank.org/72022542-DF56-4928-82F5-CCA9B224136D

##### Type material.

***Holotype***: • 1 male, slide mounted. DOMINICAN REPUBLIC, Blanco, 18°52'42.7"N, 70°30'25.7"W, 30.xi.2019, light trap, A. Höcherl, leg. (EBD I-016278). ***Paratypes***: • 1 male, slide mounted, same data as holotype (EBD I-016279). • 1 male, slide mounted, same data as holotype (LEA). • 1 male, slide mounted, Blanco, 18°52'59.9"N, 70°30'28.8"W, 01.xii.2019, CDC light trap, A. Höcherl, leg. (LEA). • 2 males, slide mounted, Blanco, 18°52'59.9"N, 70°30'28.8"W, 01.xii.2019, CDC light trap, A. Höcherl, leg. (MNHN-EJM). • 1 male, voucher, slide mounted, same data as holotype (MNHN-EJM), GenBank sequences: PV761055–PV761056. • 1 female, voucher, slide mounted, same data as holotype (EBD I-016280), GenBank sequences PV761059 to PV761060.

##### Etymology.

The species name refers to the pinkie-shaped lobe of the gonostylus (from the Latin, *digit*- (finger) and -*ulus* (small, little).

##### Diagnosis.

The new species can be distinguished from congeners in having the abdominal tergites III–IV with dark, transverse bands; a long, slender anal point; and the apex of the gonostylus slightly forked, with shallow concavity between the forks and one pinkie-finger-like lobe.

##### Description.

**Male** (*n* = 7, except when stated otherwise). Total length 2.95–3.26, 3.13 mm (5). Wing length 1.50–1.80, 1.69 mm (6). Total length/wing length 1.71–1.98 (5). Wing length/profemur length 1.77–1.90 (3).

**Figure 1.**
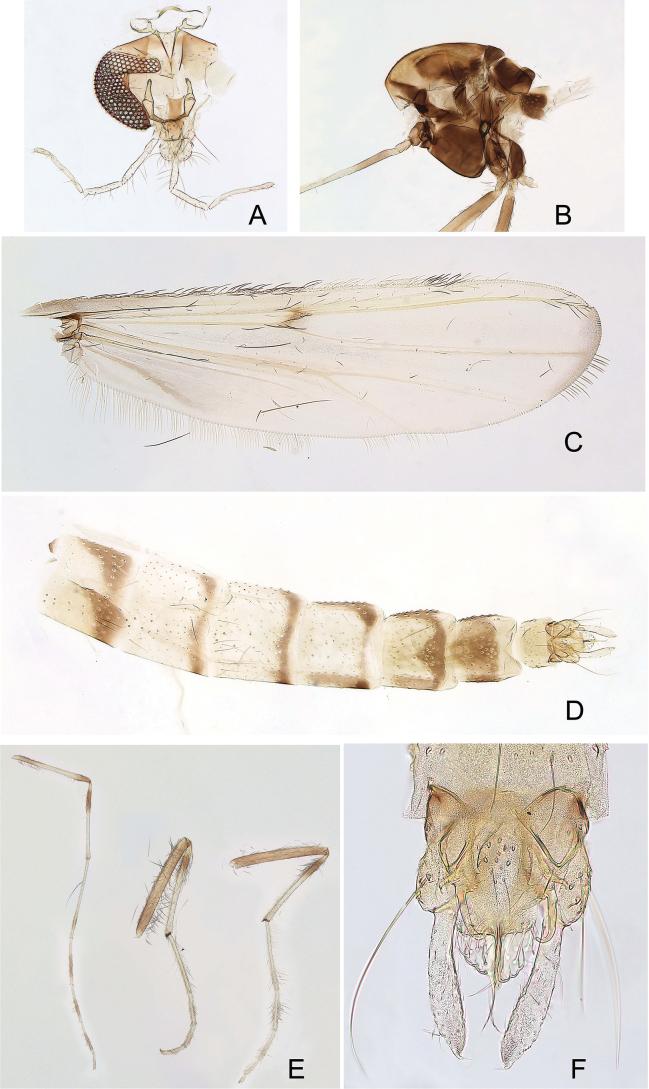
*Xestochironomus digitulus* Fusari & Baranov, sp. nov., male. A. Head; B. Thorax; C. Wing; D. Abdomen; E. Legs; F. Hypopygium.

***Coloration***: head and palp pale brown (Fig. 1A). Antenna pale brown. Thorax brownish (Fig. 1B). Halters brown. Wings with faint brownish areas on brachiolum, arculus, and crossvein RM; membrane transparent, slightly brownish along wing margin, from apex of R_4+5_ to apex of M_1+2_, and on humerus between Cu and An (Fig. 1C).

Abdominal segments coloration as follows (Fig. 1D): TI yellowish with dark-brown lateral bands; TII yellowish with dark-brown lateral and posterior bands; TIII–IV yellowish with dark-brown posterior bands; TV–VI yellowish with dark-brown lateral and posterior bands; TVII brown in anterior 2/3 with dark-brown, rectangular anteriomedial spot and posterior 1/3 of segment yellowish; TVIII yellowish.

Legs (Fig. 1E). Foreleg: femur yellowish with proximal and apical ¼ brown, tibia yellowish with 1/3 brown proximally, tarsomere 1 yellowish with brown ring proximally, tarsomere 2–4 brownish with proximal and apical ring pale, tarsomere 5 brownish with proximal ring pale. Mid- and hind legs: femur brownish, tibia yellowish with proximally ring brown and proximal 1/5 pale brown, all tarsi yellowish.

***Head*.** AR 1.2–2.0 (4). Temporal setae 9–10 (2). Clypeus with 10–18 (3) setae. Tentorium 119–139 (2) μm long. Palpomere lengths (1–5 in μm; *n* = 4): 23–36; 48–56; 124–145; 119–155; 201–242.

***Thorax*** (*n* = 5). Acrostichals 12–16, 15. Dorsocentrals 15–25, 21, partly biserial. Prealars 4–6, 5. Scutellum with 8–13, 11 setae.

***Wing*.** VR = 1.16–1.23 (5). Brachiolum with 2 (3) setae. R with 27–34 setae; R_1_ with 27–36 setae; R_4+5_ with 42–49 setae; RM with 6 setae; M with 2–3 setae apically. Squama with 7 (2) setae.

***Legs*.** Combs of midtibia 17–19 (3) μm and 26–31 (3) μm long spurs. Combs of hind tibia 21–24 (3) μm and 27–31 (3) μm long spurs. Width at apex of foretibia 46–50 (3) μm, of midtibia 41–51 (3) μm, of hind tibia 41–54 (3) μm. Lengths (in μm) and proportions of legs in Table 1.

**Table 1.** Lengths (in µm) and proportions of legs of *Xestochironomus digitulus* Fusari & Baranov sp. nov., male (*n* = 3).

	Fe	Ti	Ta1	Ta2	Ta3	Ta4	Ta5	LR	BV	SV
P1	829–949	739–918	839–971	492–555	401–458	343–393	150–174	1.04–1.13	1.73–1.86	1.87–1.92
P2	747–883	659–769	439–503	243–266	176–199	100–112	70–72	0.65–0.66	3.08–3.36	3.20–3.28
P3	857–983	796–916	626–703	362–404	296–332	167–191	84–93	0.77–0.77	2.51–2.57	2.64–2.70

**Figure 2.**
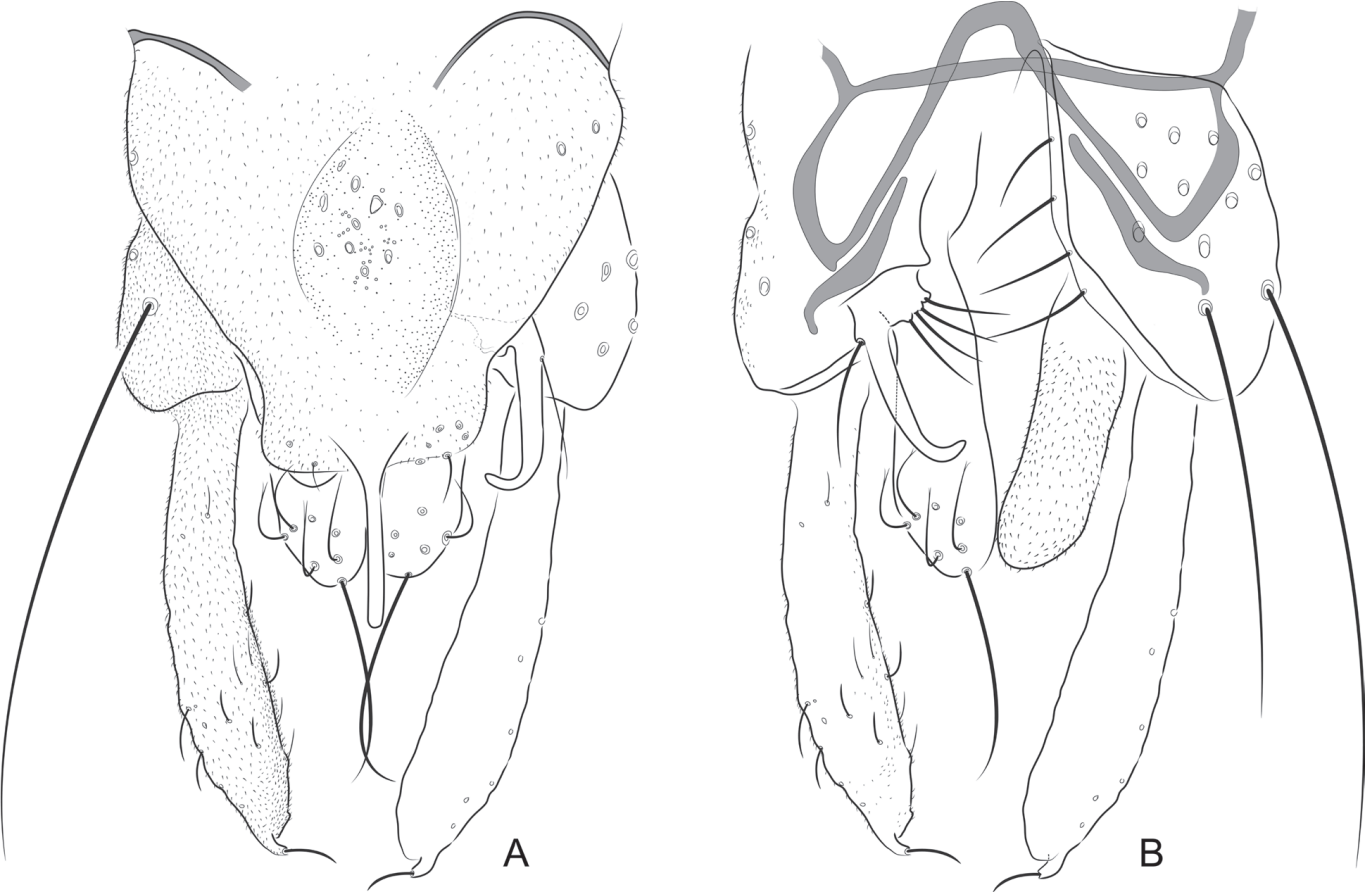
*Xestochironomus digitulus* Fusari & Baranov, sp. nov., male. A. Hypopygium, dorsal view; B. Hypopygium with tergite IX and anal point removed, left dorsal aspect, right ventral aspect.

***Hypopygium*** (Figs 1F, 2A, B). Tergite IX with 14–16 strong median setae and 6–8 weaker setae along the posterior margin on each side of the anal point, and several fine setae on the ventral side. Anal tergite bands V-shaped, separated. Laterosternite with 2–4 setae. Anal point slender, 48–59 (4) μm long, 61–65 (2) μm long in lateral position (from the base). Phallapodeme 63–73 (3) μm long. Transverse sternapodeme curved, 24–30 (3) μm long. Gonocoxite 110–135, 118 (5) μm long. Superior volsella hooked, 54–85, 79 (6) μm long, with 1 strong lateral seta and 1/3 basal setae. Inferior volsella 64–101, 91 (5) μm long, with 7–10 dorsal setae, 75–81 (3) μm long apical seta. Gonostylus bifid, 135–149, 143 (5) μm long, one pinkie-like lobes with 10–20 (4) μm long apical seta. HR 0.74–0.92 (5). HV 1.98–2.31 (5).

##### Description of female

**(*n* = 1).** Total length 2.78 mm. Wing is bent, no observations possible. Coloration similar to male.

***Head*.** Lengths of flagellomeres (μm): 80, 105, 104, 86, 104. Temporals setae 14. Clypeus with 15 setae.

***Lengths of palpomeres*** (μm). 34, 48, 136, 142, 249.

***Thorax*.** Acrostichals 12; dorsocentrals 29, partly biserial; prealar 6; scutellars 11.

***Legs*.** Combs of midtibia 20 μm and 36 μm long spurs. Combs of hind tibia 19 μm and 42 μm long spurs. Width at apex of foretibia 56 μm, of midtibia 50 μm, of hind tibia 57 μm. Lengths (in μm) and proportions of legs in Table 2.

**Table 2.** Lengths (in µm) and proportions of legs of *Xestochironomus digitulus* Fusari & Baranov, sp. nov., female (*n* = 1).

	Fe	Ti	Ta1	Ta2	Ta3	Ta4	Ta5	LR	BV	SV
P1	994	897	—	—	—	—	—	—	—	—
P2	897	752	503	232	186	108	76	0.67	3.57	3.28
P3	995	890	—	—	—	—	—	—	—	—

##### Genitalia

**Figure 3.**
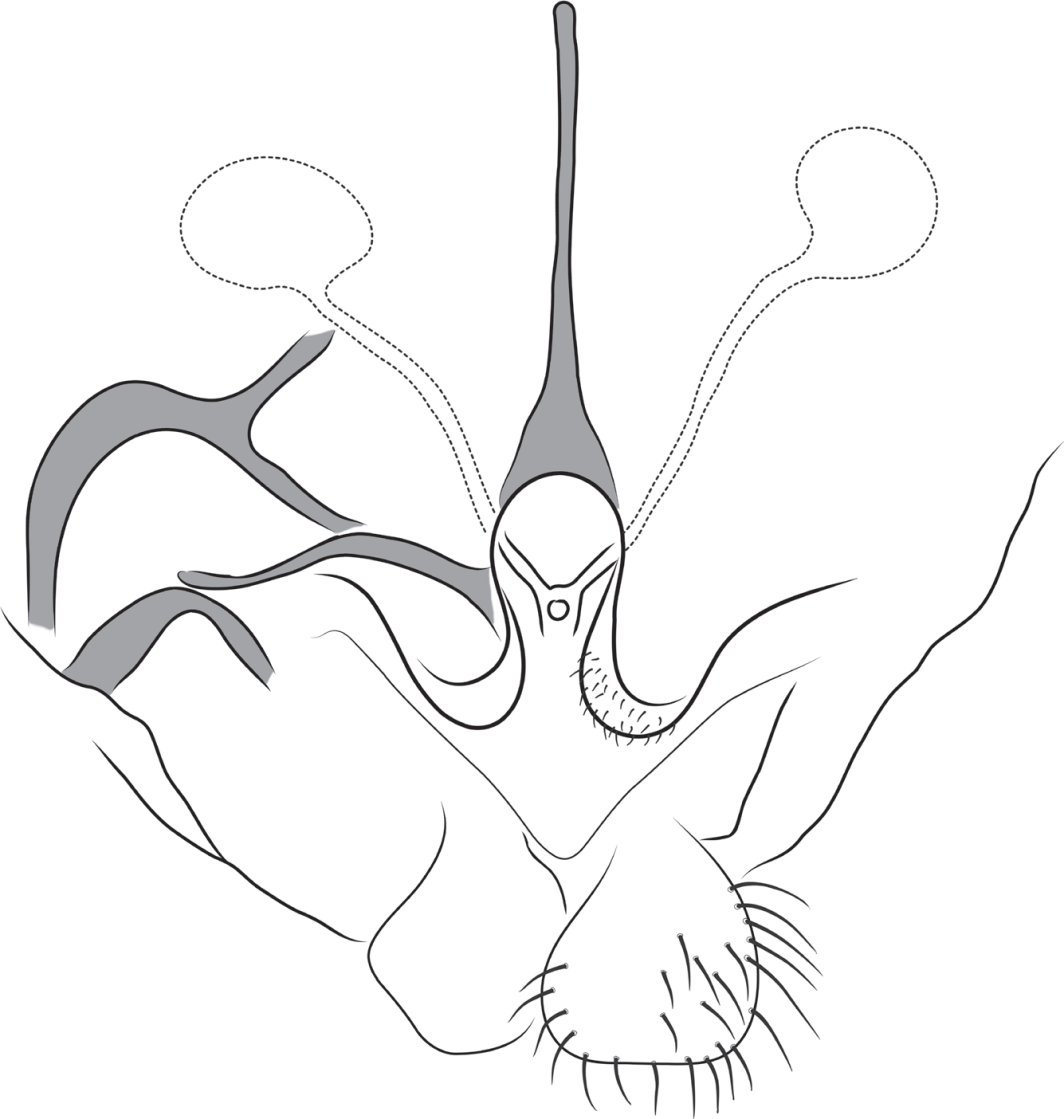
Female genitalia *Xestochironomus digitulus* Fusari & Baranov, sp. nov., left ventral aspect.

**(Fig. 3).** Tergite IX with 25 setae. Gonocoxite IX with 6 setae. Gonapophysis IX notum 236 μm long. Seminal capsules spherical, 49 μm wide. Gonocoxapodeme 225 μm long. Coxosternapodeme 134 μm long. Cercus 88 μm wide.

##### Immatures.

Unknown.

##### Distribution.

Known only from the Dominican Republic.

## ﻿Discussion

The new species belongs to *Xestochironomus* based on the combination of the following character states: anteropronutm reduced, with mesonotum protruding between lobes; tibial combs partially separated; and tip of gonostyle split into two lobes (Cranston et al. 1989). Based on the key to adult males of *Xestochironomus* (Pinho and Souza 2013) *X. digitulus* sp. nov. has a slightly forked gonostyle with a shallow concavity between the thumb-shaped lobes and an abdomen with dark, transverse stripes.

*Xestochironomus digitulus* sp. nov., *X. nebulosus*, and *X. naranjoi* have dark transverse stripes, but the new species differs from the two other species in having the lateral lobe of the gonostylus extending about ½ the length of the mesial lobe and the anal tip spatulate. In addition, the thorax of *X. naranjoi* is yellowish, with a dark-brown zigzag line posterior to the scutellum.

### ﻿ Xestochironomus luteifurcatus

Taxon classificationAnimaliaDipteraChironomidae

Sublette & Wirth, 1972

6AA5CB72-A157-5BB8-827A-F22C7E811ED5

Xestochironomus luteifurcatus Sublette & Wirth, 1972: 14, fig. 15.^1^

#### Examined material.

• 3 males (+2 voucher). Dominican Republic, Blanco, 18°52'42.7"N, 70°30'25.7"W, 30.xi.2019, light trap, A. Höcherl, leg. (EBD). • 2 males. Dominican Republic, Blanco, 18°52'42.7"N, 70°30'25.7"W, 30.xi.2019, light trap, A. Höcherl, leg. (MNHN-EJM). • 2 males, Blanco, 18°52'59.9"N, 70°30'28.8"W, 01.xii.2019, Malaise trap. A. Höcherl, leg. (LEA). • 1 female, voucher, Blanco, 18°52'42.7"N, 70°30'25.7"W, 30.xi.2019, light trap, A. Höcherl, leg. (EBD). Genbank sequences PV761057–PV761058, PV761061–PV761064.

#### Redescription.

**Male** (*n* = 9, except when stated otherwise). Total length 2.78–3.54, 3.07 mm. Wing length 1.48–1.79, 1.55 mm (8). Total length/wing length 1.97–2.15 (7). Wing length/profemur length 1.82–2.02 (6).

**Figure 4.**
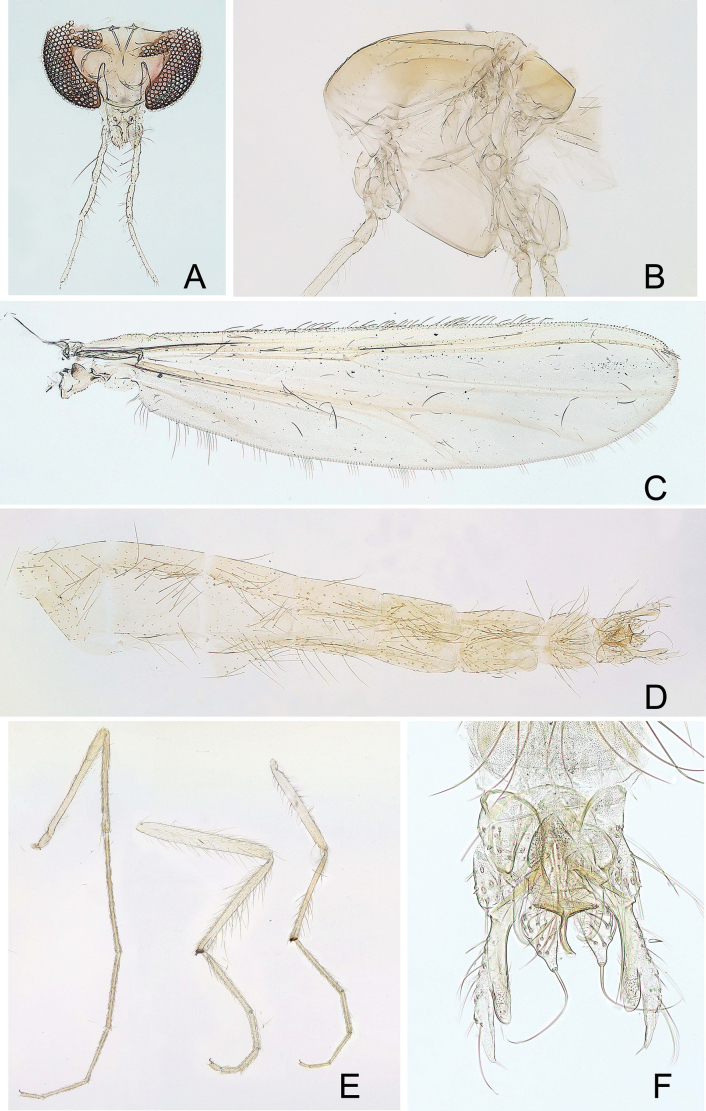
*Xestochironomus luteifurcatus*, male. A. Head; B. Thorax; C. Wing; D. Abdomen; E. Legs; F. Hypopygium.

***Coloration*.** Completely light yellow greenish and unmarked (Fig. 4A–F). Wing hyaline.

***Head*** (Fig. 4A). AR 1.31–1.47 (7). Clypeus with 6–9 (3) setae. Tentorium 114–138 (6) μm long. Palpomere lengths (1–5 in μm; *n* = 5): 30–37, 34; 39–52, 45; 124–151, 138; 119–130, 125; 201–215, 210.

***Thorax*** (Fig. 4B). Acrostichals 10–13, 12 (7). Dorsocentrals 13–29, 16 (8), partly biserial. Prealars 4 (8). Scutellum with 7–10, 9 (8) setae.

***Wing*** (Fig. 4C, *n* = 3). Venarum ratio (VR) = 1.22–1.27. Brachiolum with 2–3 setae. R with 29–31 setae; R_1_ with 20–28 setae; R_4+5_ with 37–52 setae; RM with 2–3 setae; M with 4–8 setae apically. Squama with 5–8 setae.

***Legs*** (Fig. 4E). Combs of midtibia 19–21 (4) μm and 21–22 (4) μm long spurs. Combs of hind tibia 18–19 (4) μm and 17–24 (4) μm long spurs. Width at apex of foretibia 43–4 6(4) μm, of midtibia 42–52 (4) μm, of hind tibia 48–52 (4) μm. Lengths (in μm) and proportions of legs in Table 3.

**Table 3.** Lengths (in µm) and proportions of legs of *Xestochironomus luteifurcatus*, male.

	Fe	Ti	Ta1	Ta2	Ta3	Ta4	Ta5	LR	BV	SV
P1	702–986; 814 (6)	592–839; 707 (6)	691–921 (4)	432–550 (4)	318–421 (4)	260–331 (4)	110–143 (4)	1.17–1.22 (4)	1.69–1.77 (4)	1.73–1.87 (4)
P2	622–898; 788 (6)	598–894; 706 (6)	387–673; 485 (6)	186–402; 263 (6)	150–344; 210 (6)	92–198; 127 (6)	60–92; 73 (6)	0.65–0.75 (6)	2.37–3.58 (6)	2.65–3.35 (6)
P3	634–993; 821 (6)	709–1004; 835 (6)	539–790; 636 (6)	314–452; 374 (6)	269–388; 323 (6)	144–211; 174 (6)	78–105; 90 (6)	0.76–0.79 (6)	2.37–2.47 (6)	2.49–2.66 (6)

***Hypopygium*** (Fig. 4F). Tergite IX with 10–12 strong median setae and along the posterior margin several fine setae, on the ventral side. Anal tergite bands V-shaped, separated. Laterosternite with 2–3 setae. Anal point slender, 47–66, 53 (n = 5) μm long. Phallapodeme 50–76 (2) μm long. Transverse sternapodeme, 20–28 (3) μm long. Gonocoxite 75–113, 93 (7) μm long. Superior volsella hooked, 52–86, 75 (7) μm long. Inferior volsella 64–119, 102 (8) μm long. Gonostylus bifid, 117–141, 127 (7) μm long, apex of gonostylus markedly forked, with deep concavity between thumb-like lobes. Lateral lobe of gonostylus overreaching mesial lobe. HR 0.59–0.84 (7). HV 2.38–2.69 (7).

#### Redescription of female

**(*n* = 1).** Total length 2.43 mm. Wing bent, no observations possible. Coloration similar to male.

***Head*.** Lengths of flagellomeres (μm): 52, 55, 82, 87, 111. Temporals setae 14. Clypeus with 19 setae. Tentorium = 132 μm.

***Lengths of palpomeres*** (in μm). 29, 40, 223, 152, 236.

***Thorax*.** Acrostichals 10; dorsocentrals 18, partly biserial; prealar 4; scutellars 13.

***Wing*.** Squama with 7 setae.

***Legs*.** Combs of midtibia 15 μm and 29 μm long spurs. Combs of hind tibia 16 μm and 30 μm long spurs. Width at apex of foretibia 59 μm, of midtibia 37 μm, of hind tibia 49 μm. Lengths (in μm) and proportions of legs in Table 4.

**Table 4.** Lengths (in µm) of legs of *Xestochironomus luteifurcatus*, female (*n* = 1).

	Fe	Ti	Ta1	Ta2	Ta3	Ta4	Ta5
P1	995	822	—	—	—	—	—
P2	906	800	—	—	—	—	—
P3	1006	966	—	—	—	—	—

#### Genitalia.

In a lateral position. Gonocoxite IX with 7 setae. Gonapophysis IX notum 273 μm long. Seminal capsules spherical, width 63 μm. Gonocoxapodeme with 150 μm. Coxosternapodeme with 103 μm. Cercus width 96 μm.

#### Immatures.

Unknown.

#### Distribution.

Dominica, but expanded to the Dominican Republic.

##### ﻿Barcode of the Dominican Republic *Xestochironomus*

We have conducted BLAST in GenBank. We have also conducted “Identification” query with tree-based search in BOLD v. 4. Search queries were conducted for the sequences of the five specimens of two species of Dominican Republic *Xestochironomus* species. No close matches (>85%) were found, with the closest coming from unidentified representatives of Chironomini from South and Central America. We think that the absence of the close matches is caused by the lack the *Xestochironomus* sequences in the databases (except one from Cranston et al. 2012, but that uses from different primers).

**Figure 5.**
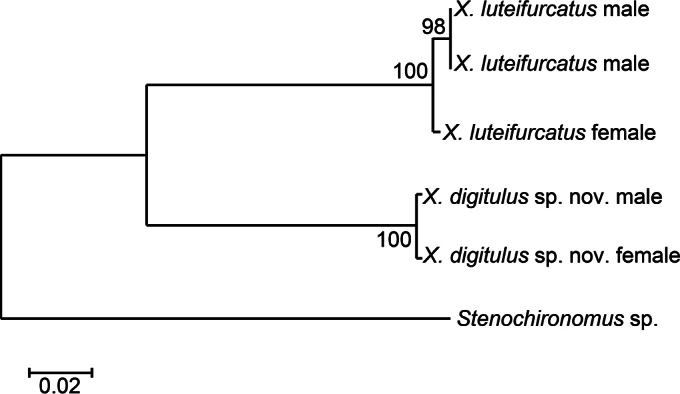
Neighbor-joining tree based on mtDNA COI sequences of *Xestochironomus* species. Bootstrap values > 50% are shown on branches.

In the neighbor-joining tree (Fig. 5), we found that the male and female of *X. digitulus* sp. nov. grouped with 99.8% bootstrap support, and that the male and female of *X. luteifurcatus* grouped at 99.2% bootstrap support. Genetic distances among the analysed species are shown in Table 5.

**Table 5.** Genetic distance between *Xestochironomus* species based on the mtDNA COI gene. Analyses were conducted using the Kimura-2-parameter model.

	1	2	3	4	5	6
1. *Stenochironomus* sp.						
2. *X. digitulus* sp. nov. male	0.272					
3. *X. digitulus* sp. nov. female	0.272	0.002				
4. *X. luteifurcatus* male (1)	0.282	0.185	0.181			
5. *X. luteifurcatus* female	0.282	0.178	0.175	0.008		
6. *X. luteifurcatus* male (2)	0.282	0.185	0.181	0.000	0.008	

## ﻿Conclusion

This study describes a new species of *Xestochironomus* and associated the male and female through DNA barcoding. It also extends the geographic range of *X. luteifurcatus* to the Dominican Republic.

## Supplementary Material

XML Treatment for Xestochironomus digitulus

XML Treatment for Xestochironomus luteifurcatus

## Footnotes

In the original description, females were not figured.

## ﻿Additional information

### Use of AI

No use of AI was reported.

### Author ORCIDs

Lívia Maria Fusari https://orcid.org/0000-0002-1107-2785

Amelie Höcherl https://orcid.org/0009-0007-4211-7468

Caroline Chimeno https://orcid.org/0000-0001-9933-6492

Viktor Baranov https://orcid.org/0000-0003-1893-3215

### Data availability

All of the data that support the findings of this study are available in the main text.
